# A Rare Case of Primary Rectal Squamous Cell Carcinoma and the Use of Cytokeratin Markers

**DOI:** 10.7759/cureus.21175

**Published:** 2022-01-12

**Authors:** Sindu Iska, Kapisthalam Kumar

**Affiliations:** 1 Internal Medicine, Regional Medical Center Bayonet Point, Hudson, USA; 2 Internal Medicine/Oncology, Regional Medical Center Bayonet Point, Hudson, USA

**Keywords:** oligometastasis, gastrointestinal neoplasm, human papillomavirus (hpv), rectal squamous cell carcinoma, chemotherapy, cytokeratin, radiation therapy, survival

## Abstract

Lower gastrointestinal cancers are commonly adenocarcinoma of the colon and rectum and squamous cell carcinoma (SCC) of the anus. Rectal squamous cell carcinoma (SCC) is a rare gastrointestinal tract malignancy, as rectal SCC is assumed to be from the migration of anal squamous cells. However, primary rectal SCC is rarer. Here, we present a case of a 63-year-old male who was found to have rectal SCC that was very close to the anus. Through literature review, it was noted that SCC and adenocarcinoma of rectal origin stain positive for cytokeratin CAM 5.2 and not the anal canal lesions. This patient's tumor was positive for CAM 5.2. The patient was treated with 5-fluorouracil and mitomycin C with radiation therapy for five weeks. The post-therapy repeat PET scan showed complete resolution of the tumor and oligometastasis. Unfortunately, the 20-week follow-up PET CT showed para-aortic and retrocrural lymph nodes consistent with malignancy. This case emphasizes the use of immunohistochemical stains for diagnosis and treatment planning in patients with rectal SCC. Once the diagnosis was confirmed, the patient was treated as anal SCC. The importance of differentiating between rectal and anal SCC can be argued, although the treatment is the same; however, the prognosis is worse based on nodal involvement in rectal SCC. Patients with early intervention have a five-year overall disease-free survival of greater than 80%.

## Introduction

Colorectal cancer is the third most common carcinoma in the United States [1]. Lower gastrointestinal cancers are commonly adenocarcinoma in the colon and rectum, whereas squamous cell carcinoma (SCC) is common in the anus. Most rectal squamous cell carcinoma (SCC) presents as a locoregional disease (stage I-III, 82.1%), and they are associated with a poorer overall survival when compared stage for stage with adenocarcinoma. The NCI states that the overall five-year survival for rectal SCC was found to be 48.9% compared with 62.1% for adenocarcinomas [2]. It is theorized that rectal SCC is mostly from the migration of anal squamous cells and hence is treated similarly with chemotherapy and radiation therapy. Primary rectal SCC is rarer, mostly seen in females with a median age of 61 years; it is known to be effectively treated with chemotherapy and radiation therapy [3].

## Case presentation

**Figure 1:**
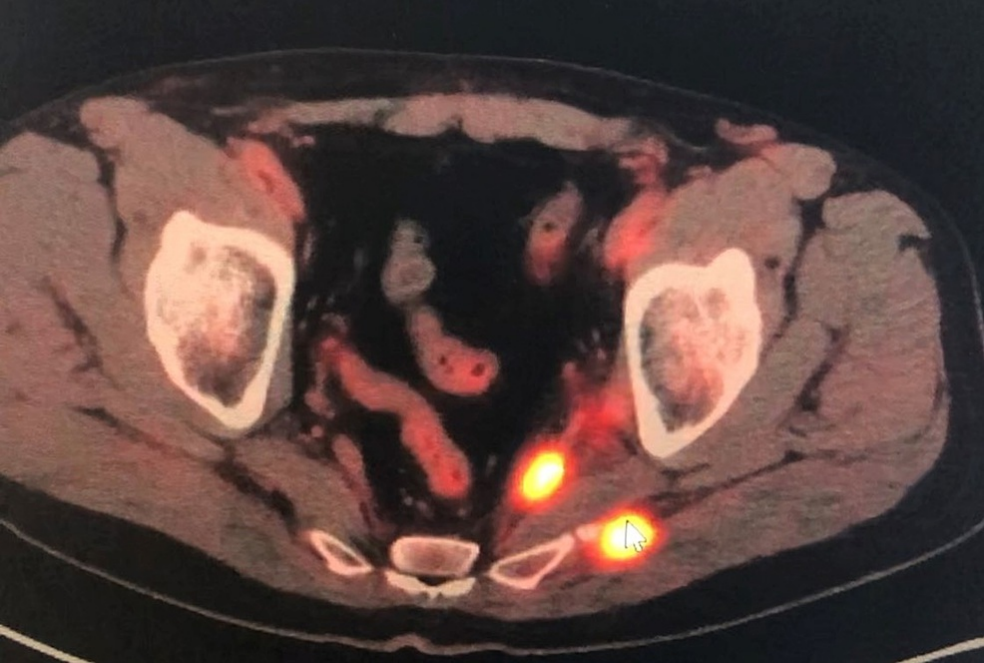
PET scan before treatment showing hypermetabolic peritoneal nodules of the left posterior pelvis, making the patient oligometastatic.

A 63-year-old Caucasian male with a medical history of transient ischemic attack, atrial flutter s/p pacemaker, and active tobacco use presented to the clinic after a recent colonoscopy result. Prior to his colonoscopy, the patient had intermittent rectal bleeding for approximately six months, which led him to see a gastroenterologist who performed a colonoscopy as a workup. The colonoscopy showed an ulcerated rectal mass in the distal rectum, 12 cm above the anal verge, measuring 2 × 2 cm. A rectal biopsy done at that time showed rectal SCC. His human papillomavirus (HPV) test was positive, and his PET scan showed hypermetabolic focus at the rectum and two peritoneal nodules in the musculature of the left pelvis (Figure 1).

**Figure 2:**
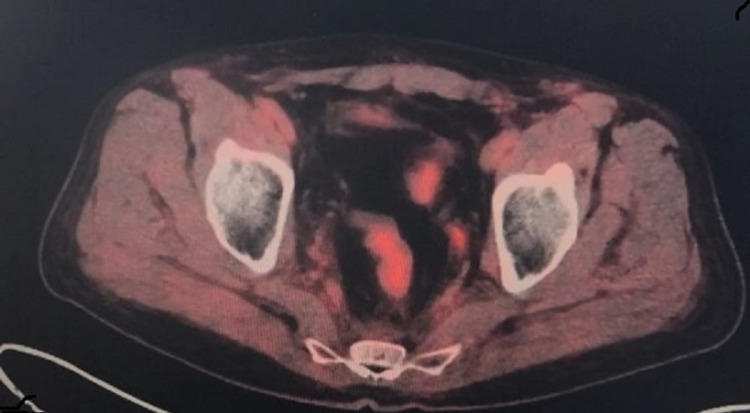
PET scan after chemotherapy and radiation therapy with complete resolution of the rectal mass and oligometastatic lesions.

He was treated as a stage 4 rectal SCC as his lesions in the pelvis were considered oligometastases. The biopsy of the lesion showed metastatic disease with poorly differentiated SCC with microfocal keratinization. Immune marker CAM 5.2 was positive in the patient. He was treated with 5-fluorouracil and mitomycin C with radiation therapy for five weeks with two cycles. This was followed by a repeat PET scan in five to eight weeks (Figure 2) that showed complete remission of the hypermetabolic focus in the rectum and peritoneal nodules.

**Figure 3:**
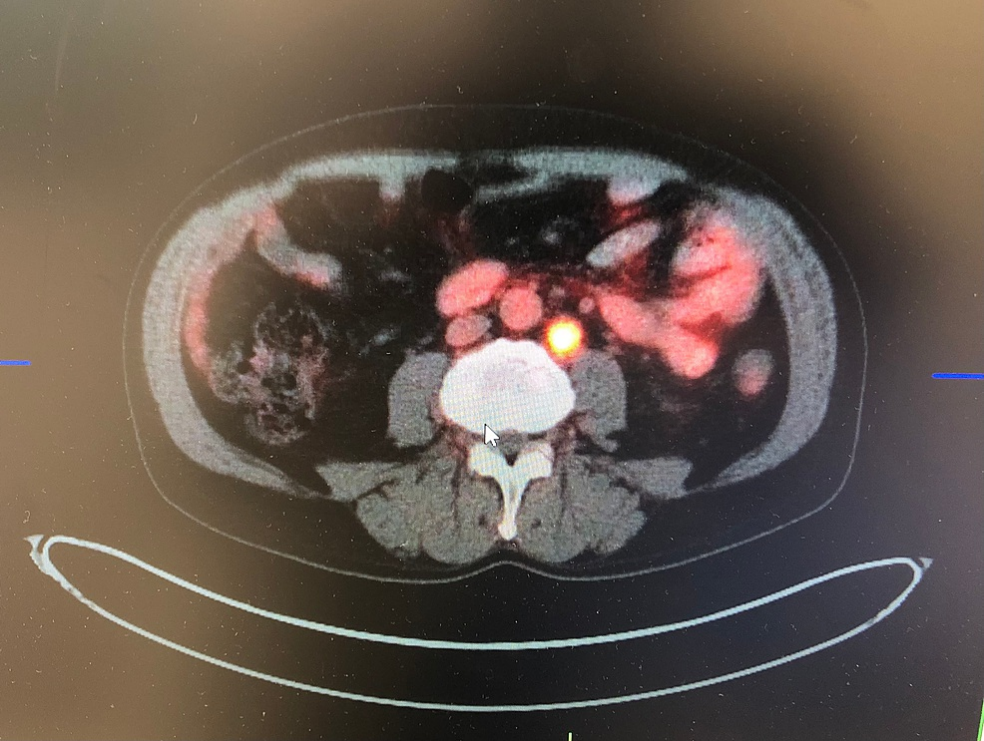
Follow-up PET CT scan 20 weeks after complete remission of the lesions showed hypermetabolic right para-aortic, left retroperitoneal, and right retrocrural lymph nodes.

The patient had a repeat PET CT scan 20 weeks after complete remission, which showed hypermetabolic right para-aortic, left retroperitoneal, and right retrocrural lymph nodes consistent with malignancy (Figure 3). He is currently on second-line chemotherapy with cisplatin and etoposide.

## Discussion

The incidence of primary rectal SCC is rising with time, and the diagnostic criteria for squamous cell cancer involving the rectum include (i) absence of evidence of squamous cell carcinoma of any other part of the body, indicating possible metastasis; (ii) careful proctoscopy/colonoscopy to exclude proximal extension of anal squamous cell carcinoma; and (iii) lack of a fistulous tract lined by squamous cells. These criteria are required in conjunction with histology to diagnose squamous carcinoma without glandular differentiation [4,5]. Through literature review and case reports, we found that most patients with SCC were treated with chemoradiation and surgery. Tumor location, markers, and histopathology will help in planning the treatment. Diagnosis relies on colonoscopy and the biopsy results of the lesion mentioned in the diagnostic criteria above. Differentiating SCC of the anus from that of the rectum can be difficult; however, it can be facilitated by immunochemical staining for cytokeratins [6]. We used CAM 5.2 to differentiate rectal from anal lesions as it stains rectal cells (squamous and adenocarcinoma), but not anal squamous cells. AE1/AE3 and p63 stain positive for squamous origin [2]. Favorable prognostic factors included SCC histology, having undergone surgery, receipt of radiation therapy, local stage, younger age, female gender, and white race [7]. Patients with rectal SCC had a better prognosis after adjusting the prognostic factors mentioned above. The importance of differentiating between the two types of cancers can be argued as, currently, they have similar treatment; however, the prognosis is worse based on nodal involvement in rectal SCC. Historically, SCC of the anal canal is treated with abdominoperineal resection until Nigro discovered that combining radiation therapy with 5-fluorouracil and mitomycin, which is fondly called the Nigro protocol, showed complete resolution of SCC of the anal canal [8], which we used in our patient with rectal SCC and was found to have complete resolution on his post-chemoradiation PET scan at eight weeks. The concomitant use of chemotherapy and radiation therapy is an effective treatment for rectal SCC with oligometastatic lesions. The term oligometastases represents a clinical state of metastatic disease that is limited in the number of metastatic sites and extent of disease, and amenable to metastasis-directed surgical or ablative therapy or radiation therapy [9]. Unfortunately, the patient failed the first-line chemotherapy and was found to have new hypermetabolic lymph nodes consistent with malignancy in his 20-week post-resolution PET CT. He was started on second-line chemotherapy with cisplatin and etoposide.

## Conclusions

Rectal squamous cell carcinoma with stage 4 disease could lead to the belief that the outcome is poor. However, we would like to emphasize the importance of precise diagnoses using immunochemical markers through this case report. Treatment with chemotherapy and radiation therapy could improve the overall prognosis of the patient; however, unfortunately, the patient had new hypermetabolic lesions on his repeat PET CT scan done at 20 weeks. We believe that if the treatment can be encompassed in the radiation field, such as in our patient with oligometastasis, patients can have a better prognosis than that predicted by published literature. We hope that in the future cytokeratin markers such as AE1/AE3, CK5/CK6, and p63 are used more often to differentiate between rectal SCC and adenocarcinoma with concurrent PET CT scan for early diagnosis and detecting recurrence. Hopefully, in the future, we can have rectal SCC-specific treatment targeting the lesion.

## References

[1] Song EJ, Jacobs CD, Palta M, Willett CG, Wu Y, Czito BG (2020). Evaluating treatment protocols for rectal squamous cell carcinomas: the Duke experience and literature. J Gastrointest Oncol.

[2] Guerra GR, Kong CH, Warrier SK, Lynch AC, Heriot AG, Ngan SY (2016). Primary squamous cell carcinoma of the rectum: an update and implications for treatment. World J Gastrointest Surg.

[3] Kommalapati A, Tella SH, Yadav S (2020). Survival and prognostic factors in patients with rectal squamous cell carcinoma. Eur J Surg Oncol.

[4] Carroll D, Rajesh PB (2001). Colonic metastases from primary squamous cell carcinoma of the lung. Eur J Cardiothorac Surg.

[5] Norris Norris, HT HT (1991). Pathology of the colon, small intestine, and anus (vol. 17).

[6] Dyson T, Draganov PV (2009). Squamous cell cancer of the rectum. World J Gastroenterol.

[7] Chiu MS, Verma V, Bennion NR (2016). Comparison of outcomes between rectal squamous cell carcinoma and adenocarcinoma. Cancer Med.

[8] Czito BG, Meyer J (2013). Radiation therapy in anal and rectal cancer. Surg Oncol Clin N Am.

[9] Milano MT, Biswas T, Simone CB 2nd, Lo SS (2021). Oligometastases: history of a hypothesis. Ann Palliat Med.

